# *Thelazia callipaeda*: infection in dogs: a new parasite for Spain

**DOI:** 10.1186/1756-3305-4-148

**Published:** 2011-07-27

**Authors:** Guadalupe Miró, Ana Montoya, Leticia Hernández, Diana Dado, María Victoria Vázquez, Marta Benito, Manuel Villagrasa, Emanuelle Brianti, Domenico Otranto

**Affiliations:** 1 Departamento de Sanidad Animal, Facultad de Veterinaria, Universidad Complutense, de Madrid, Spain; 2 Clínica Veterinaria "El Mundo Animal", Villanueva de la Vera, Cáceres, Spain; 3 Clínica Veterinaria Candeleda, Candeleda, Ávila, Spain; 4 Centro Veterinario Oftalmológico Goya, Madrid, Spain; 5 Dipartimento di Sanità Pubblica Veterinaria, Facoltà di Medicina Veterinaria, Università degli Studi di Messina, Messina, Italy; 6 Dipartimento di Sanità Pubblica e Zootecnia, Università degli Studi di Bari, Valenzano (Bari), Italy

## Abstract

**Background:**

*Thelazia callipaeda *(Spirurida, Thelaziidae), eyeworms, are known as the causative agents of thelaziosis, initially described in Asia and, later on, over the last decade, also in some European countries (e.g., Italy, France, Germany and Switzerland). In June 2010, the first case of canine thelaziosis was observed in central western Spain (La Vera region, Cáceres) and subsequent epidemiological investigation is reported in the present study.

**Results:**

This study describes the first autochthonous cases of infection by *T. callipaeda *in dogs from central western Spain where the first case of eyeworm infection was reported.

A total of 456 dogs was examined in this geographical area. *Thelazia callipaeda *eyeworms were observed in 182 (39.9%) animals, of which 28 showed apparent clinical signs (i.e., conjunctivitis, oedema, epiphora and petechiae). A total of 762 adult nematodes (214 males, 548 females; mean infection rate of 4.18; SD 4.74) were collected with cotton swabs or by flushing of the conjunctival sac of infected animals using physiological saline solution. Nematodes were identified as *T. callipaeda *according to the morphological keys and molecular analysis of sequences of a portion of the mitochondrial cytochrome c oxidase subunit 1 (*cox*1) gene. The sequences were identical to those representing *T. callipaeda *haplotype 1, previously reported in Europe.

**Conclusions:**

The high infection rate of canine thelaziosis herein reported suggests that practitioners should include this eye infection amongst differential diagnoses of ocular diseases in dogs from this area of Spain or those moving across this area of Spain. Based on the high infection prevalence recorded, the potential public health risk to humans from this region is also discussed.

## Background

Canine thelaziosis caused by *Thelazia callipaeda *(Spirurida, Thelaziidae) is an arthropod-borne disease caused by a nematode that infects, at both the adult and larval stages, the eyes of domestic (i.e., dogs and cats) and wild carnivores (i.e., foxes, wolves, beech martens and wild cats) [1] and humans [2,3]. In the infected animals, the presence of worms may induce different degrees of clinical signs, ranging from lacrimation and conjunctivitis to keratitis, epiphora, eyelid oedema, corneal ulcers and blindness [4,5]. In former times, *T. callipaeda *used to be known as "the oriental eyeworm", as its occurrence was believed to be confined to a range of Asian countries including India, Thailand, China and Japan [4]. However, following the first reports of *T. callipaeda *infection in dogs in northern Italy [6,7], canine thelaziosis had been eventually found to be endemic and highly prevalent in other areas of this country, particularly in the southern regions [8]. Most recently, autochthonous cases of thelaziosis were described in both dogs and cats in south-western France (Dordogne area) [9] and Switzerland [10] and human infections were also reported for the first time in Europe [11]. The emergence of canine ocular thelaziosis in France was demonstrated by retrospective analyses of data collected over a number of years [12], suggesting that the disease might have spread in concomitance with that of its arthropod vector. This infection is indeed known to be transmitted by the fruitfly *Phortica variegata *(Diptera, Drosophilidae), which feeds on the lacrimal secretion of the carnivore and human hosts, thus depositing *T. callipaeda *infective third stage larvae in their conjunctival sacs. Since the first demonstration of the role played by *P. variegata *as vector of *T. callipaeda *under laboratory [13] and natural conditions [14], knowledge on this nematode and its vector has gradually expanded. An ecological niche model was computed, showing that vast areas of Europe are indeed suitable for the development of *P. variegata *[15]. This evidence suggested that this infection could have spread in recent years in areas that were previously considered as non- endemic.

On June 2010, a case of canine thelaziosis was reported in an area of central western Spain (La Vera region, Cáceres) [16]. The present study describes the occurrence of autochthonous cases of thelaziosis in dogs in this area, presents clinical scenarios and provides molecular data on the genetic makeup of a representative sample of the nematodes collected.

## Methods

### Study area

**Figure 1:**
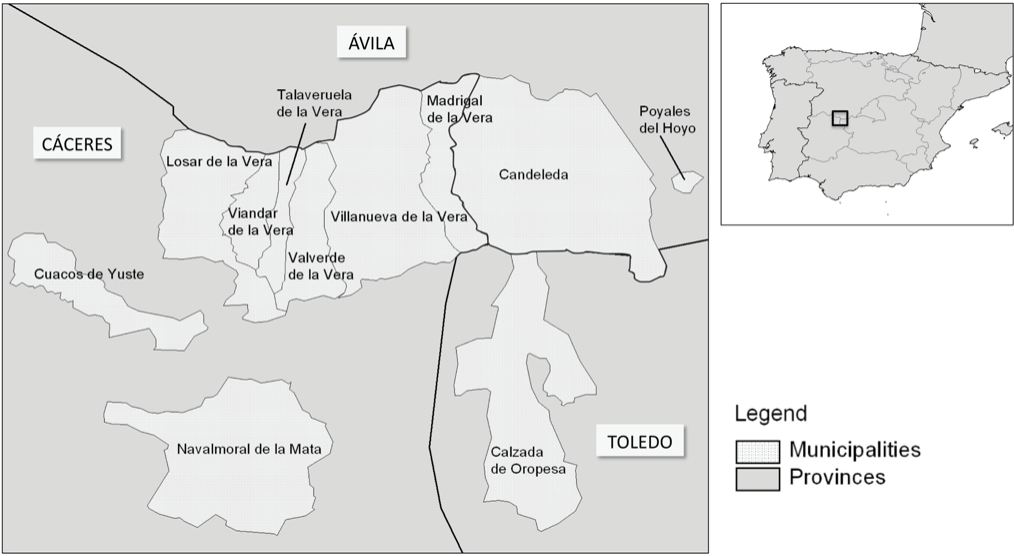
**Geographical localities where the presence of *Thelazia callipaeda *was observed**. Muncipalities: Candeleda; Madrigal de la Vera; Villanueva de la Vera; Valverde de la Vera; Taraveruela de la Vera; Losar de la Vera; Viandar de la Vera; Navalmoral de la Mata; Cuacos de Yuste.

**Figure 2:**
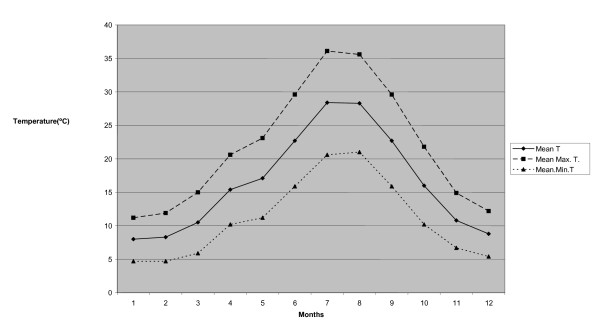
**Temperature pattern in the study area in 2010**.

The survey was carried out in seven municipalities from La Vera region (40°07'N, 5°36'W to 40°09'N, 5°14'W) in central western Spain (Figure 1). This area is characterized by mountains with valleys and river streams and vegetation mainly represented by oaks, chestnut and cherry trees. Typical crops include peppers, tobacco, raspberries and kiwi fruits. This area ranges in altitude from 250 up to 2000 m above sea level (a.s.l.) with an average altitude from 360 to 800 m a.s.l. The mean temperature recorded from May to October ranged from 17 to 30°C (Figure 2), with high relative humidity (i.e., 75-90%) and an annual average of 56.9 mm (2-162.4 mm) recorded rainfall (i.e., http://www.aemet.es).

### Clinical examination and parasite collection

From October 2010 to May 2011, a total of 456 owned dogs were examined. Data on breed, age, gender, weight (small = <15 kg; medium = 15-30 kg; and large = >30 kg), health status (including ocular signs) and municipality of origin were recorded for each individual dog. All dogs were subjected to ocular examination after administration of anaesthetic eye drops (tetracaine hydrochloride and naphazoline hydrochloride, Colircusí Anestésico^® ^0,50%). *Thelazia *spp. eyeworms were collected from the conjunctival sac of infected dogs using sterile cotton swabs or flushing with physiological saline solution.

### Morphological and molecular identification

Nematodes collected from each individual dog were identified according to the morphological keys [17] and stored in 70% ethanol before molecular analyses. Nematode specimens were subjected to specific PCR amplification of a portion of the *cox*1 (689 bp) as previously described [13]. In order to determine the haplotype/s of *T. callipaeda *collected in the present study, all amplicons were sequenced; sequences were determined in both directions (using the same primers individually as for the PCR) and the electro-pherograms verified by eye. Sequences were aligned using the ClustalX program [18] and the alignments were compared with the sequences available in public databases (i.e., NCBI at http://www.ncbi.nlm.nih.gov/) for the *cox*1 of *T. callipaeda*.

### Statistical analyses

Animal data collected were analysed using SPSS 17.0 statistical software. A Chi square analysis was used to test for associations between all of the parameters. Differences were considered significant if the *p *value was < 0.05.

### Ethical considerations

All dogs were examined by a veterinarian to evaluate any possible adverse effect related to the ocular sedation and to ensure that no dog suffered any damage during the ocular examination, according to current regulations.

## Results

Out of the 456 dogs examined, 182 (39.9%) were infected by *T. callipaeda*. Twenty (4.4%) of the positive dogs had travelled to other localities of Spain (i.e., Basque Country, Catalonia, Valencia, Madrid, Murcia and Granada) and only two abroad (i.e., one to Morocco and the other to Portugal). In all the localities abovementioned, canine thelaziosis had not been previously detected. Three dogs were housed in Madrid but they had travelled during the previous summer to La Vera region (Cáceres) (see Figure 1 and Table 1). All dogs examined spent most of their time outdoors. The prevalence of *T. callipaeda *infection was higher (p < 0.05) in large (43%) than in medium (39.3%) and small (30.1%) sized dogs, but no significant differences (p > 0.05) were recorded in infected animals when compared according to their gender or age (Table 2).

**Table 1:** Prevalence of *Thelazia callipaeda *infection in dogs, listed according to municipality.

Municipality	n (%)/Total
Candeleda	38 (30.1)/126
Madrigal de la Vera	26 (54.6)/48
Villanueva de la Vera	82 (35.6)/230
Valverde de la Vera	3 (50)/6
Taraveruela de la Vera	5(45.4)/11
Losar de la Vera	8 (100)/8
Viandar de la Vera	7 (70)/10
Poyales del Hoyo	7 (63.6)/11
Calzada de Oropesa	3 (100)/3
Madrid *	3(100)/3
	
Total	182(39.9)/456

* three dogs lived in Madrid but they had spent time during previous summers in La Vera region.

**Table 2:** Prevalence of *T. callipaeda *infection in dogs according to sex, age and body size.

	Positive
	**n (%)/Total**

Sex	
Male	99 (39.4)/251
Female	83 (40.5)/205
Age	
< 1 year	15 (23.8)/63
>1 year	167 (42.5)/393
Body size	
Small	19 (30.1)/63
Medium	64 (39.3)/163

Big	99 (43)/230

**Figure 3:**
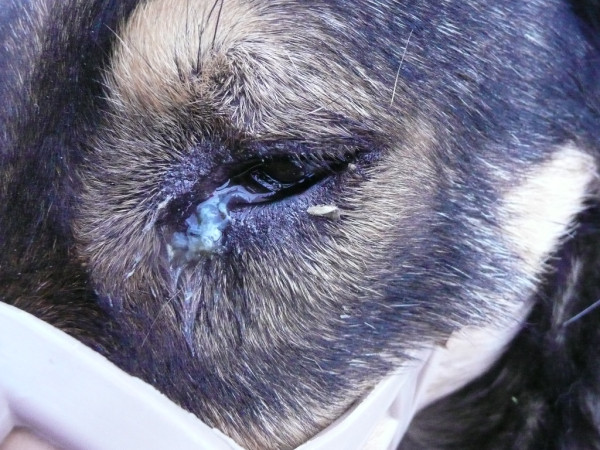
**Mucopurulent conjunctivitis in a positive dog**.

**Figure 4:**
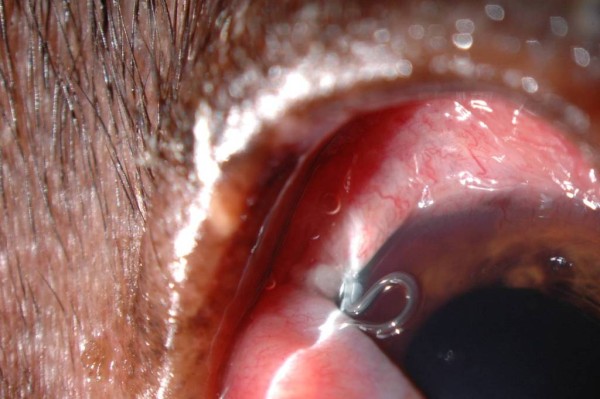
**Oedema and chemosis in a positive dog**.

**Figure 5:**
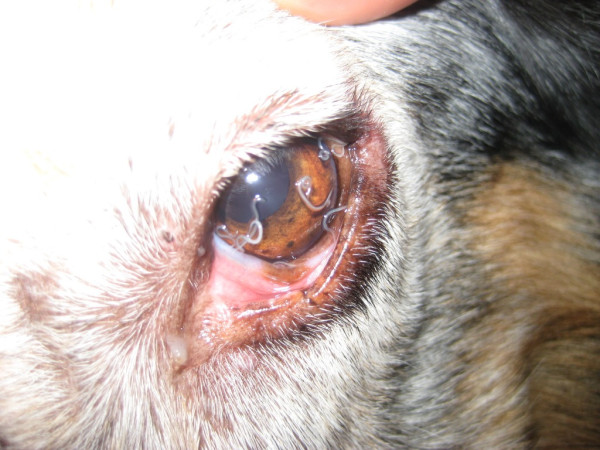
**High parasitic burden in a positive dog with conjunctivitis and oedema**.

Most of infected animals were asymptomatic (n = 154, 84.6%) whereas clinical signs of infection were observed in 28 dogs (n = 28, 15.4%). The clinical signs most frequently observed were conjunctivitis (n = 22, 78.6%) and petechiae, oedema, and epiphora (n = 6, 21.4%), respectively (Figures 3, 4 and 5). Clinical signs were not related to worm burden, the average number of worms (n = 5.87, SD = 6.7) found in dogs with clinical signs, being similar to that found in asymptomatic ones (n = 3.9, SD = 4.35).

**Figure 6:**
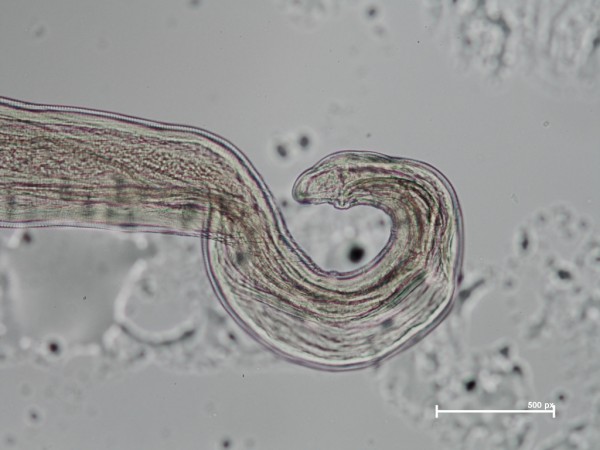
**Distal extreme of one male specimen of *T. callipaeda *(x10)**.

**Figure 7:**
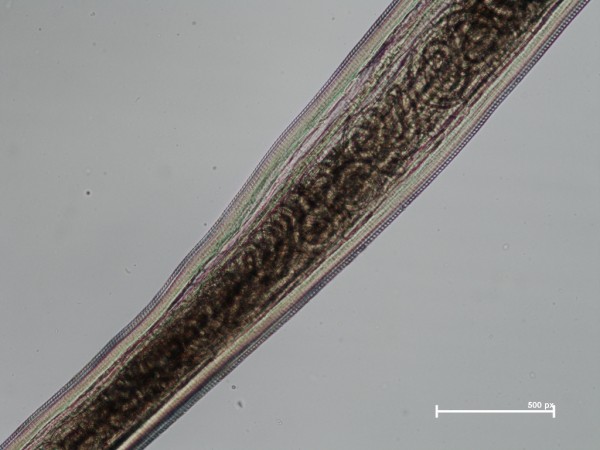


A total of 762 adult worms (i.e., n = 214 males and n = 548 females) were collected from infected dogs. The number of worms per dog ranged from 1 to 28 (4.2 ± 4.73), with only 58 dogs (31.7%) harbouring a single parasite. Of the 182 dogs with thelaziosis, 87 animals harboured only female and 15 male worms (Figure 6), the remaining 80 dogs being infected by both genders. All specimens recovered were morphologically identified as *T. callipaeda *on the basis of the presence of five pairs of large post-cloacal papillae in the ventral position in the males and of the position of the vulva, located anterior to the oesophago-intestinal junction in all females. Almost all female worms (n = 526, 95%) had intrauterine larvae (Figure 7). The *cox*1 sequences of all specimens were identical to the sequence representing h1 of *T. callipaeda *(GenBank accession number AM042549; [13]).

## Discussion

This study reports, for the first time, autochthonous cases of *T. callipaeda *infection in dogs from the central western region of Spain. All 182 animals infected came from the same geographical area of La Vera, whereas three dogs coming from a different geographical area (i.e., Madrid) had spent the previous summer along with their owners in La Vera region where, probably, they had become infected with *T. callipaeda*.

Interestingly, the latitude (40°N) of La Vera region falls within that of the European and Asian countries where cases of thelaziosis had been previously reported [8]. This area is characterized by a continental Mediterranean climate and habitat conditions similar to those described in Southern Italy where canine thelaziosis is highly endemic [8,15]. In addition, La Vera region falls within the geoclimatic provisional model for the distribution of the arthropod vector *P. variegata *[15]. Future studies focussing on the occurrence of this arthropod vector in this area would therefore desirable.

As previously reported [8,17], although infections were more frequently detected in large- sized breeds than in small ones, no statistically significant differences in the rates of infection by *T. callipaeda *were recorded. This was explained by the fact that large-sized dogs are usually housed outdoors, thus favouring physical contacts between the dogs and the arthropod vector of canine thelaziosis [10]. The percentage of dogs that presented clinical signs (i.e., 15.4%) was considerably lower than that recorded in previous studies [10,12]. This finding probably relates to the fact that dogs were examined during the early phases of infection, as suggested by the type/s of clinical signs that were observed. Although the number of worms collected from symptomatic dogs was higher than in asymptomatic ones, no relationship between the parasitic burden and the clinical signs was recorded. The molecular characterisation of the nematodes examined in the present study indicated that all *cox*1 sequences were identical to that of *T. callipaeda *haplotype 1, which had been previously detected in domestic and wild animals in Europe [13]. These data confirm the low genetic variability of *T. callipaeda *occurring in Europe and support the hypothesis that the infection could have been introduced into Spain by dogs travelling from other regions of Europe (e.g., France, Italy) where thelaziosis is endemic. Interestingly, practitioners (co-authors of this article: VV and MB) reported that during every fall season, many hunters travel together with their dogs from Italy to La Vera for game animals and that the area is also a popular destination for summer holidays, especially for French citizens. In this region, *T. callipaeda *might have found the appropriate conditions to complete its life cycle, favoured by the occurrence of both the specific arthropod vector and definitive host/s. In the same area, wild carnivores (e.g., foxes, beech martens and wolves) might act as reservoirs for this nematode and spreaders of the infection [8].

Based on the high infection prevalence recorded herein, and on the current scientific knowledge of the epidemiology of canine thelaziosis in Europe, new cases of infection are likely to emerge in other areas of Spain as well as in other European countries, both in animals and human beings. A spatial geographical model based either on the vector distribution and on the environmental and climatic features of the current endemic areas will be useful in order to estimate the spreading of this emerging parasitic disease.

Finally, canine thelaziosis should be included amongst the differential diagnoses of causes of conjunctivitis in dogs from Spain, as well as other European countries with similar environmental and climatic characteristics.

## Conclusions

This study reports for the first time the occurrence of autochthonous canine thelaziosis in La Vera region. This finding suggests that practitioners should include this eye infection amongst the differential diagnoses of ocular diseases of dogs from this area. Based on the high infection prevalence recorded, the potential public health risk to the human population from this region should also be considered.

## Competing interests

The authors declare that they have no competing interests.

## Authors' contributions

GM conceived and coordinated the study, and participated in its design, the field studies and drafted the manuscript. DO participated in carrying out the molecular assays and helped to draft the manuscript. LH and DD participated in the field studies and carrying out the diagnostic assays. MVV participated in the field studies and sampling the dogs. MB and MV participated in sampling the dogs. EB participated in data elaboration and helped to draft the manuscript. AM participated in the field studies and carrying out the diagnostic assays, performed the statistical analysis, and helped to draft the manuscript. All authors read and approved the final manuscript.
